# Ethical Dimensions of Public Health Actions and Policies With Special Focus on COVID-19

**DOI:** 10.3389/fpubh.2021.649918

**Published:** 2021-08-02

**Authors:** Basma M. Saleh, Eman Mohamed Aly, Marwa Hafiz, Rana M. Abdel Gawad, Wafa Abu El Kheir-Mataria, Mohamed Salama

**Affiliations:** ^1^Institute of Global Health and Human Ecology, School of Science and Engineering, The American University in Cairo, Cairo, Egypt; ^2^Faculty of Medicine, Mansoura University, Mansoura, Egypt; ^3^Global Brain Health Institute, Trinity College Dublin, Dublin, Ireland

**Keywords:** bioethics, COVID-19, global public health, health policy, challenges

## Abstract

During pandemics, the ethicists, public health professionals, and human rights advocates raise a red flag about different public health actions that should, at best, be addressed through integrated, global policies. How to rationalize the healthcare resources and prioritize the cases is not a recent challenge but the serious concern about that is how to achieve this while not increasing the vulnerability of the disadvantaged population. Healthcare professionals use different scoring systems as a part of their decision-making so the medical teams and triage committees can allocate resources for predictable health outcomes and prognosis as well as to appropriately triage the patients accordingly. However, the value of the existing scoring systems to manage COVID-19 cases is not well-established yet. Part of this problem includes managing non-COVID patients with chronic medical conditions like non-communicable diseases and addressing their medical needs during the pandemic complex context in a way to avoid worsening their conditions and, on the other hand, avoid hindering the establishment of comprehensive standards for dealing with COVID-19. In this article, we discuss this dilemma as well as how preexisting ethical standards were challenged by COVID-19. We also discuss how monitoring the consistent application of ethical standards during the medical trials of new medications, vaccines, or unproven medical interventions is also a critical issue.

## Health Policy Ethics, Ethical Challenges, And Opportunities

Health policy is a series of federal and non-governmental decisions and strategies to promote priorities in health care. Policies may be oriented toward people, hospitals, insurance providers, or health services. Examples include tobacco prevention policies and policies aimed at ensuring fair access to care. Policies are guided by the review of the legal powers and responsibilities of a country to make sure that people are healthy and to ensure that the country has the ability to limit the autonomy, freedom, privacy, and other legal interests of individuals for common good. These strategies must follow the ethical standards which require evidence-based public health rules based on comprehensive high-quality information on both legal actions and the optimal targets for health and well-being. Politicians typically have an ethical responsibility to ensure the rules of public health conforming to bioethics and accomplish their goals successfully. In order to identify the implications of the application of established legislation, a scientific research is continuously reviewed, and evidence is returned to the policy-making process to shape policy preferences on the current and future policies (1, 2).

The modern area of bioethics arose in the 1960's, first in North America and Western Europe and finally across the globe, in response to the enormous rise in the influence of medicine and biomedical sciences in the 20th century. Bioethics remains an important area of interest, with submissions not just from moral philosophers, but also from doctors, healthcare providers, social scientists, and lawyers. The problems of organ and tissue transplantation, differential access to life-saving drugs and new contraceptive therapies, and the significant increase in the number and kind of clinical trials are debated by health practitioners, health authorities, and government prosecutors (3). The ethics of recent health policy and system research (HPSR) largely lack a variety of normative and descriptive considerable questions. The ethical values of HPSR emphasize its commitment to reducing health inequality. Therefore, HPSR is greatly affected by legal questions regarding fairness (4).

Given the evolution of public health and the changes arising from globalization, new ethical problems have emerged in this field to pursue a coherent approach to public health regulation to deal with ethical problems. The main and classical purpose of public health is to avoid diseases rather than treat diseases. In contrast to medical practice related to patient health, the success of public health is focused on population health. The implementation of ethical standards in these two fields is distinct due to the discrepancies in clinical and public health procedures. According to public health, this sector is forever tackled by dilemmas of acceptable scope and moral conflict with personal independence in terms of its activities (5).

The application of public health data in particular communities needs information on the effectiveness, the relative damage and the gain of public health initiatives, how the initiative can be undertaken, whether it affects the most vulnerable individuals, and the logistics involved in the implementation process (6).

The need for a wider approach is gradually understood. The ideas of both public health ethics and health policy address the well-being, social rights, and collective concerns about social determinants. Debates proceed about the position of the sector, on legal functions, on rationing and the study of scenarios, on reactions to new technology, and on the healthcare systems (7).

Global health is concerned with issues of national boundaries; so solutions include global coordination and ensuring health justice for all communities, irrespective of race. It is an interdisciplinary—and multidisciplinary—system, including health concerns, explicitly or indirectly. On the other hand, international health is concerned more with problems in other suffering countries, in particular in low- and middle-income countries (LMICs); so solutions include cross-border coordination between two countries. Global health seeks to support through overlap between a variety of disciplines but it is not multidisciplinary (8).

In the massive recent pandemic we are facing, in the absence of a vaccination or effective care, people face some of the oldest and easiest ways of regulation of contagious diseases, such as quarantine, exclusion, physical separation, and construction of barriers. This condition enables one to see the value of proper diagnostic interventions, therapies, and, particularly, a vaccine as a way to restore economic and cultural life and to reunite families and friends. However, the manufacturing of a vaccine poses many public health ethics questions that are now a debate, including the implications of a quick-started search for vaccines, the equal delivery of a scarce vaccine in the beginning, and the effects of a relatively high degree of enrollment in the global immunization program (9).

**Figure 1:**
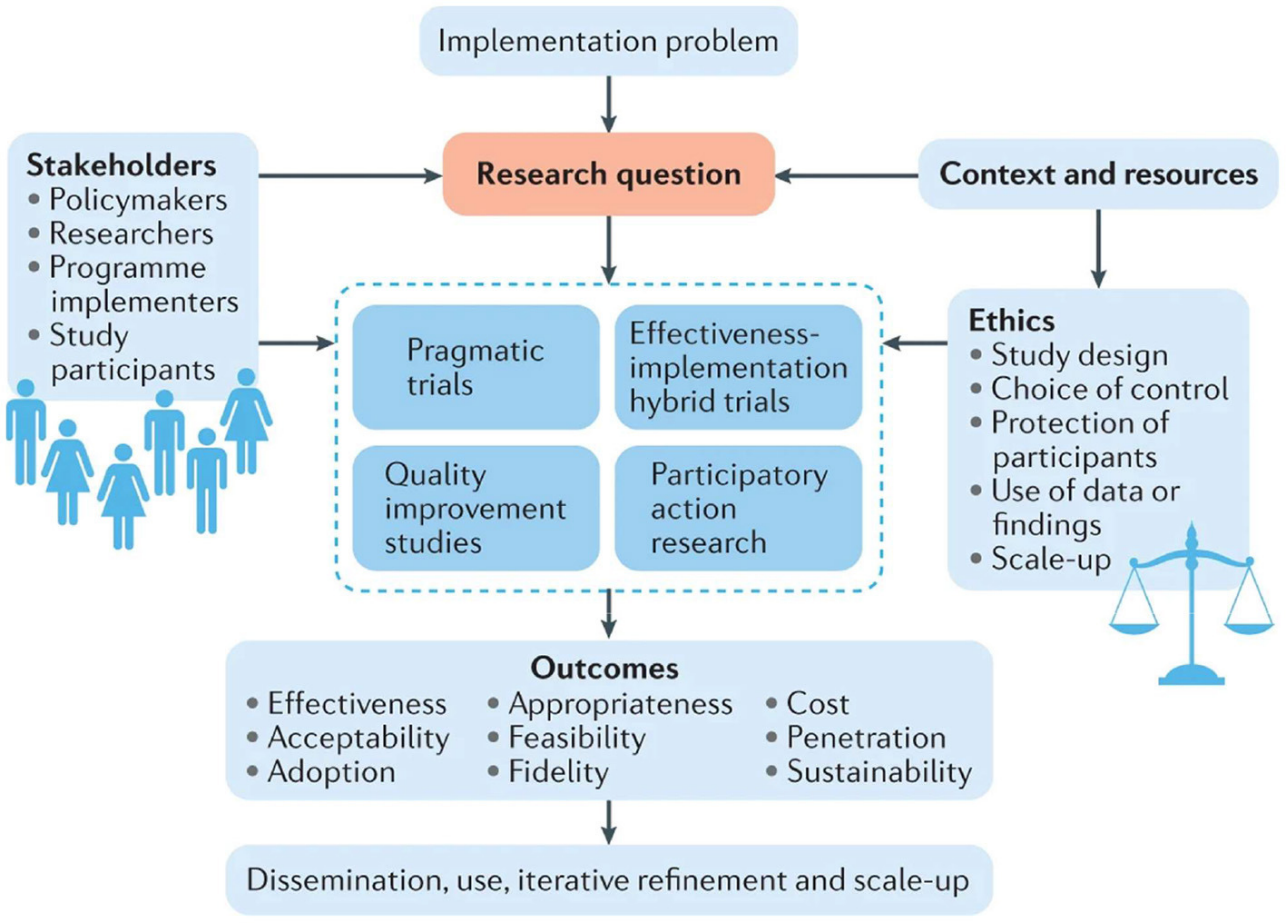
Implementation research plan for policy actions.

Although no common ethical public health theories exist, core principles and simple assumptions are common in all of these concepts. Based on the practical application of public health ethics, the implementation of appropriate concepts and standards into the policy-making procedures for public health is done through three main tasks as applied to the ethical framework: (1) to assess and explain the ethical questions posed; (2) to evaluate possible action paths and their impact; and (3) to address the issue by assessing the whole course of action by integrating the guiding principles and values and better combining them. The following four ethical principles will help in establishing the ethical recommendations: (1) ensure maximum benefit and minimum harm, (2) achieve justice, (3) eliminate inequality in health, and (4) achieve transparency (10–12). Fortunately, there is a database now for more than 200 governments responding to COVID-19 around the world from the period 1 January 2020 to 1 October, 2020, which acts as an evidence to guide policymakers and economists (13). Figure 1 discusses how we may study an arising problem of concern, utilizing the whole previous studies, trials, and research that have been done, and discussing the expected outcomes by different stakeholders, from study participants to researchers and policymakers. Continuous refinement and scaling up of the implementation plan is then done to achieve the best outcomes (14).

Ensuring an international justice lens is one of the main concepts of ethical guidelines. It is considered now—after globalization—more important than before, due to the global interdependence among countries. The health study in LMICs has been described many times as a tool for health advances to enhance global health. Benatar and Singer (15) proposed that research ethics must ensure reducing the global health gaps and promoting fairness in medical and health studies. Also harmonization of health ethics regarding policies, practices, and procedures in the countries of the area of health, particularly, countries under similar conditions like geographical distribution (16–18). This is more pronounced during pandemics as any country must be ready with its public health, healthcare, and animal programs for good pandemic planning even though the country is not considered to be at risk. It is important to coordinate with all the nations (19).

Also, providing universal health coverage is one of the sustainable development goals and it ensures that everyone enjoys healthcare without undue financial difficulties regardless of their ability to pay. There is a great gap between health coverage across regions and it is crucial to strengthen the power of health policies in regions like African regions and others with poor resources (20). Global health policymakers need to work hard and fast to implement universal health coverage, especially in the era of pandemic (21). The lack of universal access to safe and sustainable healthcare also places long-term growth opportunities at risk and exposes governments to the threats of pandemics. Without immediate actions, developing countries will be rapidly forced to close the gap between the demand for healthcare and the available public resources in order to face the aging populations and growing pressures of non-communicable diseases and will prolong the vulnerability of patients and their families (22).

Another challenge is the access to affordable high-quality medicine in LMICs, particularly in dynamically challenging conditions, despite the limited resources available for putting in place clear health strategies in those countries, and despite the fact that general health-policy research is typically driven by funds rather than needs. Non-ethical promotion activities must be regulated, their impact on access to even informal pharmaceutical markets must be considered, and policymakers must devise plans to ensure the availability of high-quality medicine (23).

Ethical obstacles for health-policy researchers, collaborating closely with stakeholders in diverse environments, to provide acceptable policy should be addressed by convening and paying attention to appropriate representation of primary stakeholders to ensure practical application of this policy in the real world. The strategy of such “partnerships” with stakeholders needs rational reflection among scientists (24).

To sum up, collective principles like utility, evidence-based performance, distributive equity, equality and social responsibility, empowerment, engagement by population, transparency, accountability, confidence, equal representation, and effectively achieving the dimension of well-being must be fulfilled. In most cases, the final ethical appraisal after evaluating the above-mentioned principles does not lead to a definitive dismissal or acceptance of public health action, but instead to a stronger or weaker decision, for example, to enforce or to forget the intervention in case of a negative assessment. In order to improve the ethical analysis and conduct of health policy and system science, it is important to gain a more detailed understanding of various applications of ethical values on both clinical study and health policy and system research by researchers and research ethic bodies. All these lead finally to creating stable social systems, empowering the talents of people, developing their thinking skills, and enhancing the autonomy based on social justice philosophy (5, 25, 26).

## Health Policy Challenges During Pandemic

One of the most challenging ethical dilemmas facing health workers and decision-makers during pandemics is rationalizing the scarce healthcare resources. High demand for intensive care unit (ICU) during COVID-19 among different populations, makes the common rule, for example, “First come, first served first” usually is not applicable in such a context. This debatable issue is not recent (27, 28). In 1960, Belding Scribner and his colleagues, at the University of Washington, developed a shunt that allows hemodialysis, three times per week, for chronic renal failure patients. Before that, hemodialysis was limited for treating acute renal failure (29). Due to the limited number of dialysis machines and trained personnel, it was essential to prioritize access to this life-long and costly treatment. In 1961, a committee composed of seven non-health professionals and two physicians were assigned for treatment prioritization.

The criteria were not restricted to medical assessment only, but also to social parameters as well. Children were excluded in favor of the heads of households who could be supporting many children. In addition, as the program was supported by the tax revenues of state, they gave priority to those patients who were residents of the state of Washington (29). In the emergency and pandemic contexts, prioritization of the patients who will benefit from access to the scarce resources as ventilators, ICU, vaccination, etc., should be justified. The process of decision-making must encounter the following principles: transparency, inclusiveness, consistency, and accountability (30). However, it is easier to say than to implement as lot of issues are involved. It is suggested that for medical assessment of the outcomes and case prognosis, prognostic scoring systems could be used to make medically justified decisions to invest more in cases with better predictable health outcomes and prognosis; for example, Sequential Organ Failure Assessment (SOFA) score for adults (31) and Pediatric Logistic Organ Dysfunction (PELOD) for children (32). Nevertheless, the predictive validity of the SOFA score in respiratory insufficiency due to COVID-19 has not been validated, in addition to the fact that it performed very well for the pandemic influenza (29). This makes it advisable to combine the scoring system with other clinical judgments until a more verified system is established.

Another ethical debatable dilemma is the right of health workers to be among the prioritized beneficiaries when they get infected. Health workers are on the frontlines in the battle against the pandemic. They sacrifice their health for the benefit of the whole society. Risking their health in limited healthcare conditions would result in a significant deficiency of health personnel who are needed to fight and respond to the emergency. Would this justify prioritizing their access to scarce resources? As a matter of fact, health workers are one of the most essential resources in fighting the pandemic. From this perspective, they are prioritized to receive medical care and protective measures, including personal protective equipment (PPE), ICUs, ventilators, and vaccines upon availability (29, 33, 34).

Moreover, patients with life-threatening medical conditions other than COVID-19 are also considered a priority for access to care. However, it is debatable if those with non-critical, non-COVID-related medical conditions should have similar access or not. While higher priority is given to patients with unstable conditions, the lowest priority goes to those patients who can tolerate health service delay until they pass the critical period of the pandemic (29).

Another argument that is not completely settled is addressing the cultural perspective in framing the regulations of resource allocation and the extent to which they should be considered (35). The argument aligns with the transparency and inclusiveness concepts of the framework recommended by WHO, putting into consideration public engagement in the drafting of such policies which would promote trust and enhance the implementation of those guidelines (36).

To conclude, it is important to establish comprehensive policy guidance for healthcare resource reallocation and rationalization in a pandemic context. This process should tackle different ethical considerations and demonstrate transparency, consistency, accountability, and inclusiveness, in which the affected community should have some degree of influence on the decision-making process. This is going to be addressed in more detail regarding COVID-19 in the following sections.

## Health Policy Challenges During COVID-19

### Resources, Medicines, and Healthcare Delivery Ethics

It is the responsibility of healthcare systems, governments, and international agencies to make sure that the best medical care possible is delivered to all in need. Unfortunately, the capacities and resources of healthcare systems are limited which create a tremendous challenge to accommodate the surging needs of population, especially in times of crisis. The current Covid-19 pandemic is a proof for such a challenge. Healthcare systems around the world are struggling to fulfill their mission. Given these circumstances, prioritization, regulations, and ethical considerations are crucial. Limited healthcare services and resources need to be allocated in a justified and ethical manner. Medical teams can be placed in a situation where they need to give priority to some patients over others in order to have the maximum benefit from the limited resources available and save many lives (37). Ventilators, medications, vaccines, and testing and admission to hospitals are the main domains of challenges, where ethical prioritization decisions are required (38).

Three main considerations can guide prioritization and decision making: first, equality between all patients in the allocation of limited medical resources. Second, prioritize patients who will benefit the most from medical resources to maximize the outcome. Medical teams can be placed in a situation where they need to give priority to some patients over others in order to get the maximum benefit from the limited resources available and save many lives. Third, allocating the resources to those with the highest medical need or at the highest risk.

In addition to these considerations, transparency, inclusiveness, consistency, and accountability are fundamentals to ensure the preservation of ethical values in resource allocation (38). There are only a limited number of COVID-19 testing kits available, although massive testing is needed to be done to obtain reliable results. So, there should be guidance on the management of COVID-19 testing. It is critical to test symptomatic patients because they will benefit from early diagnosis by receiving appropriate treatment as well as minimizing infection transmission, as most infections are known to spread through patients who exhibit symptoms. In addition, it is recommended to screen asymptomatic healthcare teams to minimize the risk of infecting patients at high risk (39).

Another service that requires prioritization is the allocation of ventilators as life-saving devices. In Italy, which suffered a major outburst of COVID-19, a guideline for ICU resources allocation was issued by the peak society for anesthesia and critical care where they suggested some limiting criteria for the use and also for the withdrawal of ventilators, like the age limit for ventilator use, and the maximum clinical benefit to be achieved (40).

Benefits can be achieved by using a clinical scoring system which focuses on saving “quality-of-life” years. Despite being an objective method, the clinical scoring system has a risk of giving a wrong precise outcome as it can give different mortality risks for patients showing the same clinical picture. As a result, the healthcare team must use the scoring system in conjunction with clinical judgment to make an unbiased and ethical decision (29). Hence, having an experienced and qualified medical team is essential in this case for maximum patient benefit (41).

Different scoring systems are available to be used by health care professionals as a part of their decision-making in times where there is a limitation to resources. The medical teams and clinicians of the triage committees must be fully aware of the exact use of each scoring system and in order to be able to select the most appropriate too. Scoring systems can be used to help physicians decide on proceeding to operate for elective cases. This scoring system needs to be continuously adapted to the rapid ongoing changes and evolution of scientific data concerning COVID-19.

Systems must be calibrated in order to establish a computerized clinical scoring system. It should then be adaptive to the continuously changing circumstances, new observations, and investigational outcomes. Scoring systems need to be finely adjusted with comorbidities as it can negatively affect vulnerable population who already suffer from diseases other than COVID-19, resulting in unfairness in limited resources allocation with these vulnerable people (42).

A study was done in many North American hospitals to compare different ventilator triage policies, and discussed that the most common principles for ventilator allocation were clinical need and patient benefit. Aside from these two principles, there were differences in ventilator allocation criteria among hospitals, which resulted in injustice. Patients can be admitted to a hospital with unfavorable policy criteria, or they can choose a hospital with favorable policy criteria; both cases are unfair. A clear ethical ventilator triage policy with specific criteria on resource allocation is essential and must be unified to maximize patient benefit and decrease any harm due to unfairness or bias (43).

It is highly important to have a triage officer with an experienced medical team in respiratory diseases responsible for making decisions to allocate limited resources and this should be totally separated from the medical care team providing treatment to the concerned patient, so as to avoid any bias or unfairness. Regular review of these resource allocation decisions by a higher-level committee should be done to avoid bias and inequities (44). In addition, this triage committee will save the treating clinicians from having to decide on whom to treat (34).

Arguments regarding the approach of the first come first served can inevitably be used in COVID-19 treatment, Nevertheless, this method can cause discrimination toward the less advantaged with poor access to healthcare facilities and who have no means of transportation (45).

Another ethical challenge for healthcare providers is to decide whether to provide non-essential healthcare services at the time of COVID-19. In several Canadian provinces, decisions have been taken to stop or reduce the non-essential medical interventions to the bare minimum until further notice, and to be substituted with telemedicine whenever possible. Healthcare providers are expected to make decisions about non-essential medical interventions based on specific criteria, including risk proportionality, the harm principle (minimizing harm), fairness, and reciprocity (mutual benefit) (46).

Moreover, due to the limited resources for patients who do not have COVID-19, standard treatment may not be available and will need to be substituted by another treatment. Most importantly, the patient must be informed by the physician of the current situation and the nature of the substitute treatment and about the type of routine treatment as well, to have well-informed consent by the patient (47).

Meanwhile, patients with non-communicable diseases (NCDs) need to have access to the needed medical care to avoid health deterioration, especially in emergency cases or cases with the need for health services done at a medical facility (e.g., chemotherapy sessions and dialysis). Healthcare entities need to provide the needed medical care for patients with NCDs separately from patients with COVID-19 to ensure the safety of those patients from getting COVID-19 infection. Telemedicine can be helpful for NCD patients who just need a consultation or follow-up of a physician (48, 49).

It should be noted that patients with COVID-19 infection should not be guaranteed to have a priority in the ICU over other patients with different diseases (45). Another important ethical debate is should health care workers be given priority in receiving medical care since they are at the front line fighting the COVID-19 and at increased risk of infection and mortality with insufficient PPE while they help many patients to survive? Should they be only given priority in preventive medical care (vaccines and medications) and not prioritized in cases that need critical care (ventilators and ICU) (29), taking into consideration that having a well-trained healthy medical team is crucial to help control a pandemic and treat COVID-19 and non-COVID-19 patients, thereby saving more lives (34).

Employers at healthcare facilities are required to provide enough PPE for the medical care team, since this will help them in taking care of patients safely and to avoid any harm (41). Furthermore, it is essential to clearly determine the responsibilities and rights of healthcare workers based on transparency and equity. If the expected benefits of public health surpass the exposed risks to health workers, then the obligation to work can be mandated (50).

Nurses are important pillars in the management of patients with COVID-19. It is crucial for the employers to protect nurses physically (providing sufficient PPE and treatment when needed) as well as legally by providing clear protocols on how to operate during a pandemic (51).

The number of nurses can affect patient recovery. As per the National Health Service (NHS), six ICU patients will require care by one critical care nurse, two nurses with ICU experience for support, another two nurses with no ICU experience, and four supporting workers. Ethical and clinical issues may arise here, such as the type of nurse-to-patient ratio required during the pandemic and whether patient prioritization is valid in terms of medical team care (41).

Meanwhile, due to the extreme load on healthcare facilities during the COVID-19 pandemic, governments need to optimize the medical work by shifting tasks, for example, using non-medical governmental staff for simple medical tasks after receiving short training (49).

A suggested solution to handle the limited medical resources is that governments can try to relocate the needed resources to areas with the highest infection rates or where the health system suffers from lack of PPEs, ventilators, or medications. Another suggestion is that retired healthcare members who are no longer in practice should get back to work and help. In addition, non-urgent medical services or procedures can be postponed without risking the lives of the patients to free up space in the medical system to be able to accommodate emergency cases (52).

Medical staff reallocation can be done to help with managing the COVID-19 pandemic workload. Reallocating staff should be guided by continuing to manage urgent cases, maintaining a mitigation plan, and providing the basic medical care services needed to avoid abandoning any patient, which is an ethical rule. This should be routinely reviewed in light of the updated circumstances and with complete transparency with patients. Meanwhile, the medical staff relocated to new roles must be given the needed training, support, and be fully prepared for the new role (53).

Medical and ethical laws in each country must be followed. For example, in Egypt, it is not allowed to deny a patient the right to receive a life-saving medical service and to be replaced by another patient whose survival chance is higher (54).

Moreover, although online consultations between physicians and patients are done to maintain social distancing and to avoid infection transmission, laws regarding online diagnosis and follow-up without a physical examination by the treating doctor must be reviewed since online diagnosis is not allowed in some countries, like Egypt (54).

To conclude, the main challenge arising in the COVID-19 pandemic is the limitations of medical resources (ICUs, ventilators, medications, PPEs, doctors, and nurses). Harmonized policies must be set to deal with this ethically to avoid bias and unfairness to patients.

### Addressing Inequities in COVID-19

Health inequity is defined as “those inequalities in health that are deemed to be unfair or stem from some form of injustice.” Thereafter, health inequalities can be regarded as avoidable inequalities (55). In the current COVID-19 pandemic, inequity is a major ethical concern. Inequities in exposure, vulnerability, and consequences of the virus are evident. The main causes of these inequities are the disparities in social determinants of health, such as income, gender, and ethnicity. These disparities lead to inequities in exposure to the virus, vulnerability to the virus, and its consequences (56).

Individuals with lower income are usually poor, with a lower level of education, and live in crowded areas with poor or no sanitation services (57), which results in a higher incidence of COVID-19 among them (58, 59). The poorest quintile of the population of LMIC is estimated to have a 32% higher risk of death from COVID-19 than that of the wealthiest quantile (60).

As for gender, men and women face different challenges due to COVID-19. Several studies have concluded that men have higher COVID-19 mortality rates (61, 62). This can be attributed to the institutionalized gender roles as well as to culture and norms which influence the risk of men with COVID-19 infection and their accessibility for testing (63). On the other hand, other studies have indicated that COVID-19 affects women differently. COVID-19 has increased the care burden on women (64), gender-based violence (65, 66), and unemployment, especially for mothers (67).

Ethnicity is also found to be a determining factor. Ethnic minority groups around the world are also disproportionately affected by COVID-19. Many studies performed in different countries have indicated such inequities (68, 69). Furthermore, the increased pressure on health systems during COVID-19 raised concerns about the existing increase in inequities with regard to access to health services by marginalized population, which extends to health services not related to COVID-19, such as organ transplantation and end-of-life care (70).

Thus, with COVID-19, it has become evident that in order to address the current situation, two considerations are to be taken into account: first, health inequities are at the root of the problem and need to be considered through all policies. Second, health ought to be central to all policies. Health is affected by policies across all different sectors, but it also affects other sectors. Therefore, considering the health aspect while formulating public policies is fundamental (71). On the other hand, policies that address health inequity in the context of COVID-19 are indispensable. All policies need to aim at addressing present inequities while providing a solution to the current crisis. Certain populations, due to their socioeconomic conditions, which are a result of public policies, cannot adhere to certain public health measures, such as social distancing or doing their jobs from home. Therefore, health policies should be backed up with the support of public policies, such as food security programs and unemployment insurance that can enable these populations to adhere to public health measures (72). Different countries have addressed health inequalities revealed in COVID-19 by adopting various types of equity-enhancing policies. Countries have adopted extra measures to reach their most vulnerable populations. In China, inequity is manifested in the difference in medical insurance scheme benefit coverage. The government has addressed this inequity by providing the hospitals with extra insurance funds to cover all admitted patients so that they do not need to pay anything regardless of their insurance scheme (73, 74).

Moreover, public health policies ought to concentrate on prioritizing high-risk communities in testing and treatment. Finally, adopted policies are to be evaluated depending on the equity criteria (75).

Despite the above arguments concerning health equity as a cornerstone in all policies proposed to face COVID-19, some professionals debate that these policies on COVID 19 are to be based not only on equity aspects, but also on two other aspects, such as the scarcity of resources and the value of intervention. The ICUs and ventilator accessibility are two examples of resources that are allocated based on the availability and benefit value. The medical professionals argue that admitting these patients to the ICU and placing them on ventilators will prolong their suffering, causing harm rather than benefit. They also argue that if equity were the admission criterion, these patients would be admitted at the expense of other patients with a higher chance of recovery (76).

Equity at the global level is another concern regarding addressing the COVID-19 crisis. An ethical global leadership is required to promote solidarity actions, protect the right of the humans with regard to health and ensure equity (77). Policy formulation, scientific knowledge production, and the current vaccine distribution are the most pronounced areas of concern (78). Policies based on using an intersectoral approach are required to address the structural and socioeconomic disparities leading to inequities in COVID-19 (79). As for the scientific knowledge, there is a need for gender and ethnically disaggregated data for the preparedness and response phases of COVID-19. These data enable scientists to determine the effect of COVID-19 on gender and ethnic bases (80, 81). Finally, there is an equity concern about the current COVID-19 vaccine distribution. Although several entities and pharmaceutical companies still work on developing more vaccines for COVID-19, many developing countries fear that high-income countries will use the Advance Purchase Agreements to guarantee full coverage of their national needs for the vaccine while leaving other nations deprived. As an initiative to avoid inequities caused by vaccine nationalism, COVID-19 Vaccine Global Access (COVAX) was established. COVAX uses the funds of the donors to buy a guaranteed vaccine supply for Low and Middle-Income Countries (82).

### Bioethics and Regulatory Challenges Regarding the Use of Unproven Interventions During COVID-19 Pandemic

The breakout of the coronavirus pandemic in December 2019 in Wuhan, China, has caused a massive influence on China and the world (83). Healthcare systems all over the world have been severely stressed and the high infection rate of the novel coronavirus imposed a serious public health threat menacing the safety of the people and hugely impacting their life norms. Due to the severity of the disease, the WHO declared the outbreak by the end of January 2020, a “Public Health Emergency of International Concern.” The world had to face an unprecedented, ruthless pandemic with limited knowledge and understanding of the disease and the virus. Several scientific studies have been conducted to achieve a more precise and full picture of the contingency and prognosis of the coronavirus. However, until now, no proven effective treatment for the management of the coronavirus has been discovered (83, 84).

Under normal conditions, the provision for new interventions for the treatment or prevention of diseases passes through a rigorous process to ensure their safety and efficacy before authorization. Usually, this is carried out by the National Regulatory Authority (NRA) and involves several procedures, starting from primary testing through research studies to produce generalized knowledge, proper allocation, and security of participants, thus leading to the proper collection of reliable data (85). Randomized controlled trials (RCTs)—specifically, the blinded and double-blinded—constitute the cornerstone for new drugs and authorization of medical interventions. RCTs are considered to be the gold standard for research studies by regulatory authorities, which ensure the appropriateness of the design to produce credible scientific evidence, protect the rights, safety, and privacy of human participants and apply sufficient measures to prevent data falsification and fraud. Based on the governing regulations adopted by different countries, the control and clearance of scientific trials considers scientific and ethical aspects that fall under the duties of various bodies and institutions, such as research ethics boards, national regulatory authorities, and ministries of health (86). These entities work to establish protocols and research procedures in alignment with local, regional, and international guidelines addressing the principles laid by the International Council for Harmonization (ICH) of Technical Requirements for Pharmaceuticals for Human Use (87), the Declaration of Helsinki (88), Pan American Health Organization (PAHO), the International Ethical Guidelines for Health-related Research Involving Humans proposed by the Council of International Organizations of Medical Sciences (CIOMS) (89), and the good clinical practices proposed by WHO (86). However, in COVID-19, as similar to previous outbreaks, the urgency to rapidly produce safe and efficient interventions for the prevention and treatment of the virus has allowed for limited deviation from the research protocols of clinical trials involving human participants (90, 91). This covers various types of interventions extending from the proposed blood components (e.g., convalescent plasma), approved drugs (e.g., Ivermectin), investigational new drugs that have not been previously authorized for the treatment of any medical condition (e.g., Remdesivir), and off-label uses of drugs authorized for the treatment of conditions other than COVID-19 (e.g., Hydroxychloroquine) (85).

### Regulatory Amendments for the Research on COVID-19 Clinical Trials

By the end of January 2020, WHO and PAHO have issued temporary recommendations for the confinement of the outbreak through the promotion of diverse public health measures. These measures included continuous investigations to recognize the epidemiology of the virus, its outbreak source, progress, transmission potential, and possible maneuvering measures to control it. The recommendations also emphasized the impact of global collaboration on the advancement of scientific knowledge about the virus and the disease through active participation and multisectoral collaboration, parallel to the promotion of the research for the containment of the disease and for the development of efficient and safe therapeutic and preventive interventions. In addition, it pointed to the crucial adherence to the ethical guidelines whenever unauthorized interventions are exceptionally offered beyond research settings (83, 86, 92, 93).

As of 28 November, 2020, and in response to the COVID-19 outbreak, the number of registered clinical trials has reached 4,029, while, on the other hand, the number of confirmed worldwide COVID-19 cases has jumped to 61.9 million (94). The design of the majority of trials varies from epidemiological investigations to single-arm studies, real-world studies, cross-sectional studies, and cohort studies, while rigorous double-blind randomized clinical trials account for a limited number due to resource deficiency and treatment pressures (83). It is well-acknowledged that ethical reviews by the Ethical Review Committee (ERC) are mandatory for any biomedical or clinical research involving human participants. However, in an infectious disease outbreak, the conduction and design of clinical trials deviate from the normal track, driven by the magnitude and contagiousness of the disease, limited time, and over-stressed health systems. Ethical committees in outbreaks are required to balance between individual interests and common public benefit when seeking results with careful consideration of the rights of the participants and prioritizing life (83). Nonetheless, routine conduction of ethical reviews will negatively affect the prompt necessity in epidemics while expedited reviews could be criticized for tactical concerns (95).

Maintaining the privacy protection of research participants would reduce the burden on researchers and balance the risk/benefit ratio, leading to more effective conduction and benefit of trials. Accepting oral informed consent and deferring the signed written form until after recovery would reduce the risk of infection during the research. Data could then be withdrawn in case of the signing refrainment of later participants (83). In addition, ERC must carefully assess the condition of the research site for the sample size required and human resources, since the high competition for the recruitment of subjects between similar trials that test the same hypothesis yet, incapable of conveying robust results due to drawbacks in the design and execution, minimizes the chances of testing another different hypothesis (96, 97). It must also take into account the balance between collective and individual interests and general ethical concerns when evaluating informed consents and risk/benefit ratio since in pandemics, health care personnel face major challenges in fulfilling their daily medical obligations along with their scientific research duties, such as observations, obtaining consents, and updating data records (83, 98). Furthermore, alleviating the harmful effects of the pandemic critically relies on fast responses and productive collaboration between ERCs which can be achieved using virtual meetings, video conferencing, and teleconferencing technologies to improve the communication and coordination and permit electronic archiving of documentation of REC (99, 100). Also, adopting a reduced quorum strategy, electronic signatures, and tighter committee deadlines would facilitate the conduction of review meetings and speed up decisions (83).

Moreover, the implementation of multinational and multicentral trials is highly recommended in pandemic as it facilitates cross-comparison with different treatment options, allows for better-coordinated evaluation, lessens the time needed to satisfy the number of participants required to generate valid conclusions, and enable adaptations of specific countries. On the other hand, adjusting to regulatory regulations of various countries, securing official approvals, and organizing among multiple personnel present challenges to the effective execution of multicenter trials (101). Nonetheless, registration of clinical trials during pandemic is mandatory, as it identifies the clinical trials that have been conducted and their specific findings. This is achieved through both national registries and by the International Clinical Trials Registry Platform (ICTRP) of WHO. Also, the development of one specific REC within each country that undertakes the responsibility of all clinical trials related to COVID-19, is highly advisable (86).

### Monitored Emergency Use of Unregistered and Experimental Interventions

Following the Ebola outbreak in 2014, when almost 30,000 humans developed the Ebola virus, the WHO formulated the “Monitored Emergency Use of Unregistered and Experimental Interventions” (MEURI), in response to the encountered extraordinary challenges (85, 102). It focuses on the exceptional access to unproven interventions outside clinical research, provided that sufficient regulatory and ethical management is applied while contributing to the generalization of scientific evidence (85, 93). The MEURI framework identified the criteria of the conditions under which the provision of unproven interventions would be considered ethically appropriate and summarizes it as follows (103):

▪ When immediate initiation of clinical trials is not feasible.
▪ In the absence of proven effective and successful treatments.
▪ Upon the availability of supportive preliminary data on the safety and efficacy of the intervention and its use outside clinical trials has been proposed by a qualified consultative scientific committee.
▪ When MEURI use has been approved by a qualified research ethics committee and by the health authorities of countries.
▪ Upon the availability of reasonable resources for risk minimization.
▪ When monitoring the use of interventions is achieved and proper, transparent, and accurate documentation and sharing of the outcomes among national and global stakeholders are adopted promptly and without delay.

In “Expanded Access” programs—formerly named “Compassionate Use” programs—innovative medications with some relevant evidence of possible effectiveness are provided to seriously sick patients who cannot take part in clinical trials and lack access to other efficient therapeutic options. Although the use of unproven interventions under “expanded access” guidelines could be ethical during pandemics, they must not compromise efforts or resources needed to conduct clinical trials which provide scientific-based evidence for the efficient and safe authorization of innovative interventions (86). The correlation of MEURI to specific regulatory frameworks, such as the “expanded access,” the lack of familiarity with the MEURI guidelines, unclear boundaries that differentiate between research intervention and MEURI intervention, are some examples of the challenges created by the MEURI framework. Also, the absence of sufficient regulatory and ethical control, such as the prior approval of an ethical board would put the integrity of the consent forms and the generalization of evidence in question. Furthermore, the deficiency of appropriate justification for the use of the MEURI framework in exceptional conditions during pandemics may lead to its abuse (85). Fairness, minimizing harm, maximizing benefits, and autonomy are the main key principles for the justification of MEURI intervention use (104). Recommendations for better MEURI enactment during pandemics involve limiting the time frame under which MEURI interventions are offered to prevent resource diversion from necessary clinical trials, immediate switch to clinical trials whenever possible, empowering ethical and regulatory management, a special distinctive registry of MEURI interventions, and enhancing community engagement to emphasize the continuous monitoring process and encourage risk/benefit open dialogues (85).

To conclude, the high progress rate of pandemics has forced the development of new knowledge acquisition tools other than the standard clinical trial protocols. This is made possible through continuous renovation and reformation of regulatory rules and the constant improvement of the capacity of REC. Beneficence, equity, transparency, responsibility, autonomy, and efficiency must be balanced against the best therapeutic evaluations in clinical trials, and support for accessible, reasonably effective, and adequately safe therapeutic interventions with approved participant informed consent and under the management of a clinician should be guaranteed.

## Conclusion

Health policies shape the entire health care landscape, including both patients and providers, where the best interests of both categories should be considered. In this regard, comprehensive and clearly defined bioethical standards should be embedded in drafting these policies, especially during pandemics. Over several decades, the world has experienced various health crises, from which public health professionals and ethicists have learned to build on and try to prepare for the next one. However, the COVID-19 outbreak reveals a lot of questions and defines regulations that need to be revised and adjusted. During pandemics, rationalizing the limited resources of the health-care system is a multifaceted decision that includes many pillars that policymakers should consider when developing international guidelines. One of the main aspects is setting specific measures to prioritize cases *via* medical assessment and predictive outcomes. This should be clearly stated, be evidence-based, and all the health workers assigned to triage should be informed about these measures. The second aspect is prioritizing the health workers who are on the frontline of fighting the pandemic, providing them with the necessary support to be able to perform their duties efficiently. This includes the provision of PPE, training if needed, and timely information sharing, as required. The third aspect is that there should be a clear guidance of triage for patients who do not have COVID, and managing their health care needs according to the urgency of their cases. The fourth aspect is ensuring bioethical considerations in using unproven medical interventions, unregistered medications, and experimental procedures. To ensure a more efficient implementation of the policies produced, all of these aspects should involve the participation of different stockholders, undertaking transparency, and taking cultural differences into account. Furthermore, policies and guidelines for pandemic health responses should ensure that the provision of health services and triage does not exacerbate any kind of inequality, whether they are gender-based, ethnic-based, religious-based, or other discrimination.

## Author Contributions

BS designed the concept. BS, EA, MH, RA, and WE contributed equally to conceiving, drafting, and reviewing the manuscript. MS supervised the work. All authors contributed to the article and approved the submitted version.

## Conflict of Interest

The authors declare that the research was conducted in the absence of any commercial or financial relationships that could be construed as a potential conflict of interest.

## Publisher's Note

All claims expressed in this article are solely those of the authors and do not necessarily represent those of their affiliated organizations, or those of the publisher, the editors and the reviewers. Any product that may be evaluated in this article, or claim that may be made by its manufacturer, is not guaranteed or endorsed by the publisher.
